# A Highly Mechanical, Conductive, and Cryophylactic Double Network Hydrogel for Flexible and Low-Temperature Tolerant Strain Sensors

**DOI:** 10.3390/gels8070424

**Published:** 2022-07-07

**Authors:** Quan Diao, Hongyan Liu, Yanyu Yang

**Affiliations:** 1 College of Materials & Chemical Engineering, Zhongyuan University of Technology, Zhengzhou 450007, China; 2 College of Materials Science and Engineering, Zhengzhou University, Zhengzhou 450001, China; liuhongyan@ipe.ac.cn

**Keywords:** hydrogel, double network, mechanical property, freeze-resistant, strain sensor

## Abstract

Due to their stretchability, conductivity, and good biocompatibility, hydrogels have been recognized as potential materials for flexible sensors. However, it is still challenging for hydrogels to meet the conductivity, mechanical strength, and freeze-resistant requirements in practice. In this study, a chitosan-poly (acrylic acid-co-acrylamide) double network (DN) hydrogel was prepared by immersing the chitosan-poly (acrylic acid-co-acrylamide) composite hydrogel into Fe_2_(SO_4_)_3_ solution. Due to the formation of an energy-dissipative chitosan physical network, the DN hydrogel possessed excellent tensile and compression properties. Moreover, the incorporation of the inorganic salt endowed the DN hydrogel with excellent conductivity and freeze-resistance. The strain sensor prepared using this DN hydrogel displayed remarkable sensitivity and reliability in detecting stretching and bending deformations. In addition, this DN hydrogel sensor also worked well at a lower temperature (−20 °C). The highly mechanical, conductive, and freeze-resistant DN hydrogel revealed a promising application in the field of wearable devices.

## 1. Introduction

With the increasing demands of pressure and deformation detection in artificial intelligence devices, strain sensors have attracted massive attention in recent years [1,2,3,4]. A strain sensor can detect a subtle tensile and bending deformation by outputting electrical signals such as current, resistance, and capacitance changes [5,6,7]. For better application in human body detection, strain sensors based on soft and biocompatible materials need to be developed. Unlike ordinary polymers such as plastic and rubber, hydrogels, which consist of a 3D-polymeric network swelling in water, have the advantages of softness and biocompatibility. Therefore, the hydrogel can be used in tissue engineering, flexible electronics, and soft robots [8,9,10,11]. In addition, the conductivity, mechanical strength, and freeze-resistant properties are the essential factors for strain sensors [12,13,14,15].

Due to the crystallization of internal water molecules, the hydrogel’s performance becomes poor in sub-zero temperatures. One effective way to improve the low temperature performance of hydrogels is incorporating organic solvents, such as glycerol [16], ethylene glycol [17], and dimethyl sulfoxide (DMSO) [18]. For instance, Rong et al. reported that the strain sensor made by H_2_O/ ethylene glycol organohydrogel could work in sub-zero temperatures, such as −40 °C [17]. The incorporation of ethylene glycol was beneficial for generating hydrogen bonds with water molecules and impeding the formation of ice crystals. Li et al. developed the DMSO/H_2_O system, which contained polyvinyl alcohol, cellulose nanofibers, and AlCl_3_. The DMSO in the system formed hydrogen bonds with water molecules and effectively extended the operating temperature (−50 °C or 50 °C) [18]. However, some organic fluids, such as DMSO and ethylene glycol, are not as ecofriendly as water [18,19]. Moreover, ionic liquids also have been used to prepared conductive gels with a broad range in their operation temperature [20,21]. Notably, the introduction of inorganic salts is the most economical method for enhancing the freeze-resistant property of hydrogels. Suo et al. proposed a freeze-resistant alginate-Ca^2+^/polyacrylamide double network (DN) hydrogel that could maintain stretchability, toughness, and conductivity at temperatures far below the freezing point of water [22].

Currently, many strain sensors based on hydrogels have been devised. Most hydrogels are given electrical conductivity with conductive fillers (carbon materials [23], inorganic salts [24], metal nanoparticles [25], etc.). Among these fillers, inorganic salts can disperse in the hydrogels homogenously without phase separation [26]. The water in the hydrogel dissolves salts, and the network structure provides migration channels for the dissociated ions. Additionally, the ions can combine with the hydrogel network through hydrogen bonds or coordination bonds, which can lower the freezing point of the hydrogel. Meanwhile, the dissociated ions can be utilized to build a newly formed network with additives in hydrogels. The generated network provides the hydrogel with stronger mechanical properties [27]. Inspired by this, the highly mechanical DN hydrogels were candidates for flexible strain sensors [28,29,30,31].

**Figure 1:**
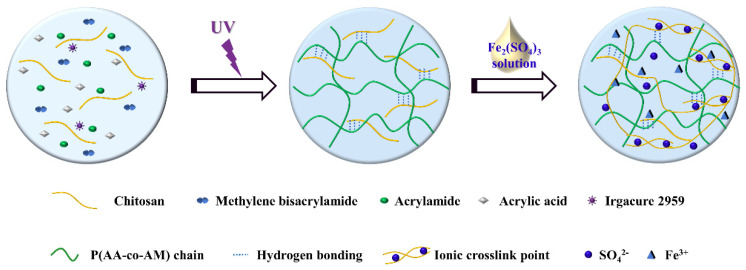
The schematic diagram of the preparation of CS/P(AA-co-AM) DN hydrogel.

In this study, we fabricated a DN hydrogel with excellent stretchability, conductivity, and cryophylactic properties. As shown in Figure 1, this chitosan-poly (acrylic acid-co-acrylamide) (CS/P (AA-co-AM)) double network hydrogel comprised of chitosan with an ionically cross-linked network. The DN hydrogel was prepared by soaking CS/P (AA-co-AM) composites into an Fe_2_(SO_4_)_3_ solution. The prepared DN hydrogel exhibited outstanding mechanical properties: remarkable stretchability (ε ≈ 400%) and compressibility (ε = 98%), and excellent anti-fatigue performance. The strain transducer made by this DN hydrogel showed prominent sensing performance for tensile deformation: the response (defined as $\frac{R-R_{0}}{R_{0}}$) was 4.92 at a strain of 500%. Moreover, the low-temperature property of the prepared transducer was also good: the response was 1.15 (at the tensile of 180%) at −20 °C. Generally, the study offers a potential material for monitoring the human body’s movements.

## 2. Results and Discussion

### 2.1. Mechanical Properties of Hydrogels

**Figure 2:**
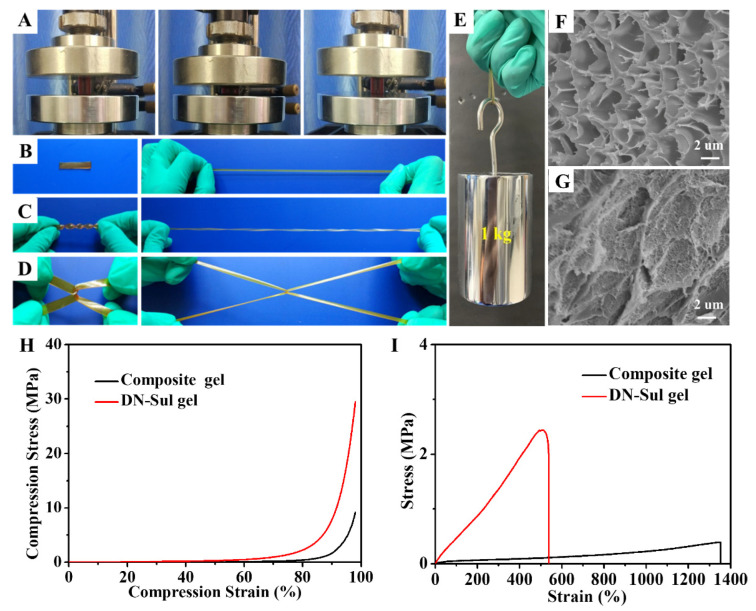
The mechanical properties of the DN hydrogel. (**A**) Compression, (**B**) stretching, (**C**) twisting–stretching, and (**D**) crossing–stretching. (**E**) Load-bearing ability (loading weight: 1 kg). SEM images of (**F**) the composite hydrogel and (**G**) the DN hydrogel. (**H**) Compressive and (**I**) tensile curves of the composite and DN hydrogels.

As shown in Figure 2A, the DN hydrogel withstood rigorous compression and recovered quickly without any deformation. In addition, the hydrogel could bear several stretching behaviors, such as direct stretching, twisting–stretching, and crossing–stretching (Figure 2B–D). The hydrogel also withstood a load of 1 kg without cracking (Figure 2E). The tensile strength and toughness of the DN hydrogel were clearly superior to that of composite hydrogel (Figure S1). The prominent mechanical performance of the DN hydrogel was due to the synergic effect of the internal dual network: one was the poly(hydroxyethyl acrylamide) network derived from the photo-initiated crosslinking of AA and AM, while the other was the ionic network of CS and Fe^3+^. The SEM images (Figure 2F,G) exhibited the comparison of micromorphology. The DN hydrogel showed denser microstructure compared with the composite hydrogel due to the formation of the CS ionic network.

The detailed mechanical properties were measured by compression and tensile tests. At 98% compressive strain, the compression stress of DN hydrogel reached 29.59 MPa, while the value of the composite hydrogel was 9.23 MPa (Figure 2H). Remarkably, the DN hydrogel remained intact during the whole compressive process, demonstrating its superior compressibility. Meanwhile, the DN hydrogel also exhibited an improved tensile strength of 2.43 MPa, which was six times that of the original composite hydrogel (Figure 2I). Figure S2A shows that the elastic modulus of the DN hydrogel reached 0.5 MPa, much larger than that of the composite hydrogel (0.03 MPa). The toughness was calculated by the area integration of the stress–strain curves (Figure S2B). The toughness of the DN hydrogel (6.79 MJ/m^3^) was triple that of the composite hydrogel (2.18 MJ/m^3^). In addition, the fracture strain of the DN hydrogel was 537%, as shown in Figure 2I. As reported, when the physicochemical DN hydrogel was deformed, its physical bonds broke and dissipated energy to protect the chemical bonds [32,33]. At the first stage of elongation, the dynamic physical interactions (CO_2_LFe^III^ and hydrogen bond) together with the chitosan physical network were destroyed for dissipating energy. Once the tensile strain exceeded 537%, both the physical and covalent cross-links were ruptured.

**Figure 3:**
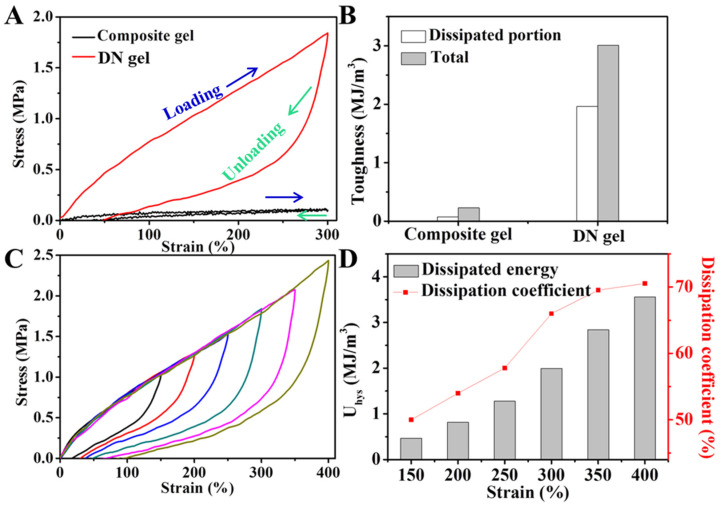
(**A**) Loading–unloading curves of composite and DN hydrogels at a strain of 300% and (**B**) their corresponding calculated dissipated energy and energy-dissipation coefficient. (**C**) Loading–unloading curves of DN hydrogel at different strains (150%, 200%, 250%, 300%, 350%, and 400%) and (**D**) corresponding calculated dissipated energy and energy-dissipation coefficient.

Figure 3A shows the energy-dissipation capacity of the hydrogels measured by a stretching–restoring test. The area of the loading–unloading curve of the composite hydrogel was very small, and the loading curve and unloading curve almost coincided. The hysteresis loop area of the ion–covalent DN hydrogel was larger, and the dissipation energy was 1.96 MJ/m^3^ by integration (Figure 3B). This was attributed to the fact that the chitosan’s physical cross-linking network and ionic coordination (CO_2_LFe^III^) provided an effective energy-dissipation mechanism for the DN hydrogel. The energy-dissipation capacity of the DN hydrogel with different tensile strains (150–400%) is shown in Figure 3C. It was found that the hysteresis loop area was aggrandized with the hydrogel’s deformation increase. The dissipation energy increased from 0.47 MJ/m^3^ at the tensile strain of 150% to 3.56 MJ/m^3^ at a 400% tensile strain, while the dissipation coefficient increased from 50% to 70.5% (Figure 3D).

**Figure 4:**
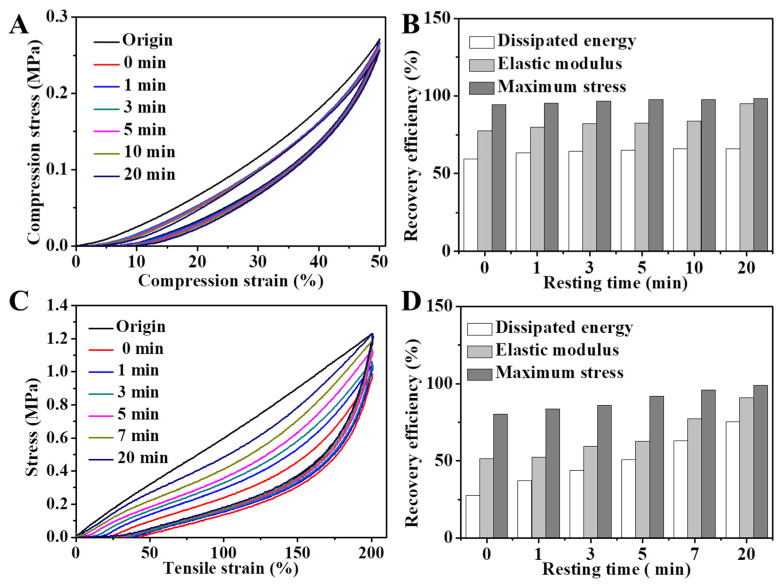
(**A**) Self-recovery behavior and (**B**) self-recovery efficiency upon compression. (**C**) Self-recovery behavior and (**D**) self-recovery efficiency upon elongation.

Figure 4A,B show the DN hydrogel’s compression self-recovery ability. During the test, six DN hydrogel specimens were compressed at a 50% strain and then left to rest for different amounts of time (0, 1, 3, 5, 10, and 20 min) before being recompressed. Compared with the original sample, the dissipated energy of the 0 min specimen remained 59.5%, with remarkable elastic modulus (77.7%) and maximum stress (94.5%). After resting for 20 min, the elastic modulus reverted to 95%, with the maximum stress restored to 98.4%, very close to the initial state. In addition, the tensile self-recovery ability was also tested as the specimens were stretched to 200% deformation (Figure 4C,D). After 20 min, the dissipated energy of the stretched sample recovered to 75.4%. Meanwhile, the elastic coefficient and tensile strength basically recovered, and the recovery efficiency was 91.5% and 98.5%, respectively. These tests demonstrated the eminent self-restorability of the DN hydrogel after compression and tensile deformation.

**Figure 5:**
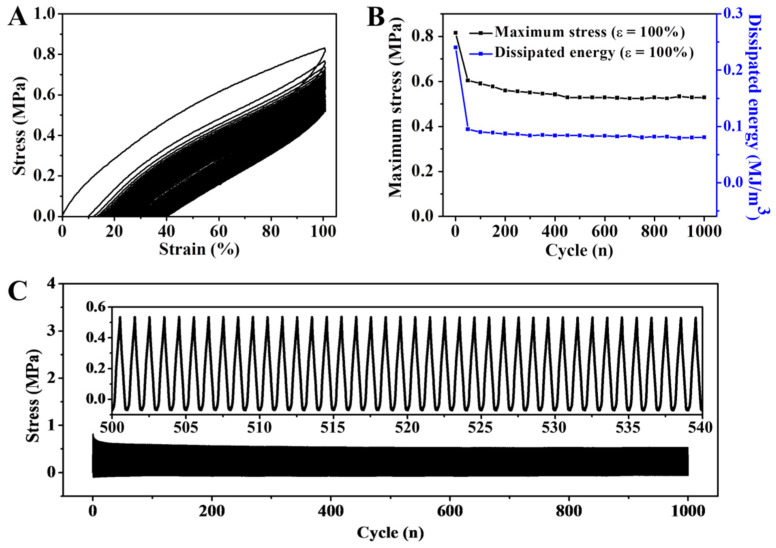
(**A**) 1000 continuous elongation–relaxation cycles of the DN hydrogel. (**B**) The evolution of maximal stress and dissipated energy during 1000 cycles. (**C**) Real-time stress curve during a continuous 1000 cycles at a strain of 100%. The built-in figure was the stress curve during the 500th to 540th cycles.

In order to further study the stability and anti-softening properties, DN hydrogel was encapsulated with 3 M adhesive tape to avoid the loss and evaporation of water during the testing process. The hydrogel sample was then subjected to a continuous loading and unloading cycle test 1000 times (ε = 100%). As shown in Figure 5A, the stress and dissipation ring area tested in the first cycle were larger than those of the subsequent ones, and the maximum stress was 0.83 MPa. It was found from the stretching curve of the 2nd to 1000th cycles that the mechanical properties of DN hydrogel were relatively stable and essentially unchanged. As shown in Figure 5B, according to the maximum stress and dissipation energy of 1000 tensile cycles, the values of the 2nd to 1000th tensile cycles were basically the same, which strongly demonstrated the stability of the mechanical properties of DN hydrogel. As shown in Figure 5C, the 1000th tensile cycle curve was locally amplified during the 500th to 540th cycles. The maximum tensile strength basically remained at the same level, indicating that the DN hydrogel had not only ultra-high mechanical strength but also stability.

### 2.2. Modulation on Mechanical Performance of DN Hydrogel

**Figure 6:**
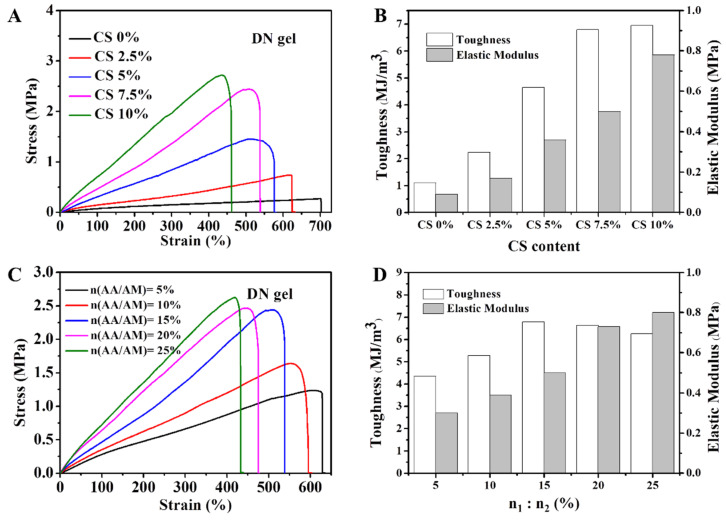
(**A**) The stress–strain curves of the DN hydrogels with various CS content and (**B**) their corresponding toughness and elastic modulus. (**C**) The stress–strain curves of the DN hydrogels with various molar ratios of AA to AM (n_1_:n_2_) and (**D**) their corresponding toughness and elastic modulus.

Due to the ionic coordination between CS and sulfate solution, the increase of CS content directly affected the CS ionically crosslinked degree and changed the rigidity of the physical network. Thus, the CS content could be used to regulate the mechanical properties of the DN hydrogel. As shown in Figure 6A,B, when the molar ratio of acrylic acid to acrylamide was 15%, the concentration of ferric sulfate was 2 M, and the soaking time was 50 min, the hydrogel’s rigidity was increased, elastic modulus enlarged, ductility reduced, and toughness enlarged as the CS content increased (the detailed data of the mechanical properties of the DN hydrogels with different CS contents are listed in Table 1). According to the influence of the chitosan content on the mechanical properties and considering the tensile strength and ductility of the hydrogel, the final fixed CS content was 7.5%. In addition, the molar ratio of the soft and tough chemical network can also affect the mechanical properties of hydrogels. Therefore, by adjusting the molar ratio of acrylic acid to acrylamide, the mechanical properties of hydrogels could be regulated. As shown in Figure 6C,D, when the molar ratio of acrylic acid to acrylamide increased, the tensile strength of DN hydrogel gradually increased, the ductility decreased, and the elastic modulus increased (the detailed data of the mechanical properties of the DN hydrogels with different molar ratios of AA to AM are listed in Table 2). Taking into account the toughness, the hydrogel prepared with 7.5% CS content, and 15% acrylic acid was the optimal sample.

### 2.3. Mechanical Flexibility at Low Temperatures

**Figure 7:**
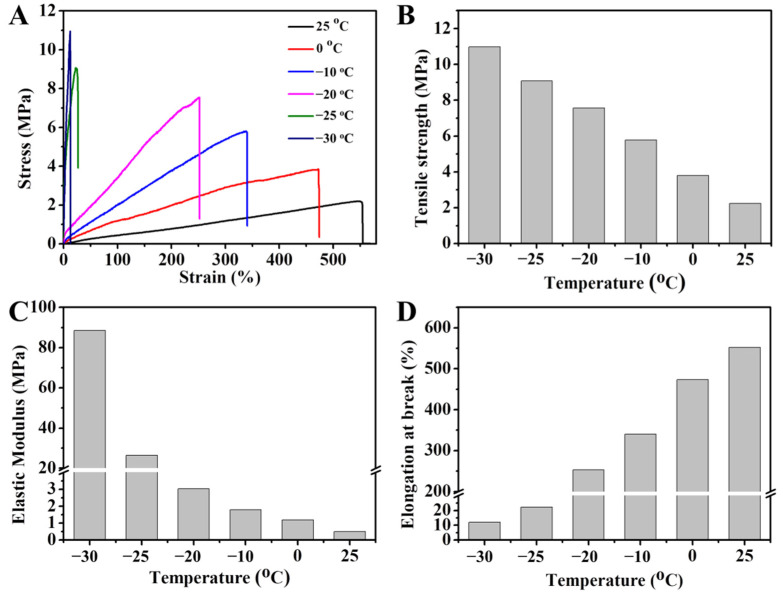
(**A**) The stress–strain curves and the detailed mechanical property parameters of the DN hydrogel at different temperatures: (**B**) tensile strength, (**C**) elastic modulus, and (**D**) fracture strain.

Because the water inside hydrogels tends to crystallize below 0 °C, the mechanical properties of the hydrogel will be damaged. Incorporating salt in the hydrogel is an effective way to depress the ice point [22,34,35]. Compared with the composite hydrogel, the DN hydrogel still maintained good mechanical properties: after being stored at −20 °C for 24 h, the DN hydrogel could be stretched and bended, while the composite hydrogel became stiff and easy to crack (Figure S3). In addition, to study the cryogenic mechanical properties of the composite and DN hydrogel in more detail, the tensile tests were made at low temperature. When the temperature went down to −20 °C, the breaking elongation of the composite hydrogel decreased to 28% (Figure S4A). However, even at −20 °C, the DN hydrogel still maintained good mechanical properties, with 250% breaking elongation (Figure 7A). As the temperature dropped below −20 °C, the gel converted into a mixture of ice crystals and polymer networks, leading to reduced flexibility and ductility, together with enhanced rigidity [36]. The detailed mechanical properties parameters are illustrated in Figure 7B–D and Figure S4B–D. As the temperature decreased, the tensile strength and elastic modulus of the hydrogels were aggrandized, whereas the breaking elongation decreased rapidly. The Fe^3+^ and ${\mathrm{SO}}_{4}^{2-}$ were expected to prevent water molecules from gathering and crystallizing, resulting in the outstanding freeze-resistant property.

### 2.4. Sensing Performance of Hydrogel Flexible Sensor

**Figure 8:**
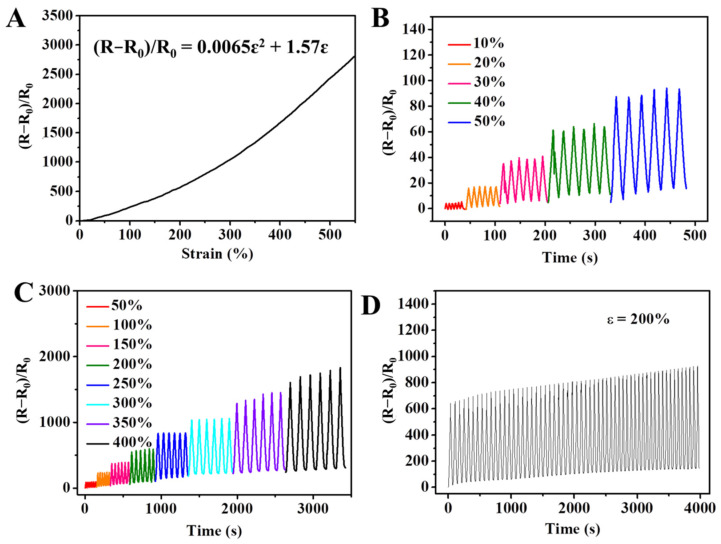
The performance of the DN hydrogel sensor: (**A**) relationship between resistance variation and tensile strain; (**B**,**C**) relative resistance-variation curves of multiple tensile strains; (**D**) real-time resistance-variation curves within 60 cycles (ɛ = 200%).

In addition to the outstanding mechanical properties, the ions in the DN hydrogel also provided good ionic conductivity, which led the hydrogel to transmit electric signals through ionic migration. The conductivity of the hydrogel varied with the type of deformation, such as stretching, compression, and twisting. Therefore, the hydrogel can be used in flexible sensors. As shown in Figure 8A, the hydrogel exhibited a resistant-type behavior to the tensile deformation; as the resistance increased, the tensile strain continued to grow. The hydrogel sensor exhibited good sensitivity to a small strain (10–50%) and large strain (50–400%), as shown in Figure 8B,C. As the previous literature reported, the gauge factor (GF) of the sensor can be calculated from the curve of the relative resistance variation ($\frac{R-R_{0}}{R_{0}}$) and strain [37]. In detail, the relationship between $\frac{R-R_{0}}{R_{0}}$ and strain (ɛ) can be turned into a formula: $\frac{R-R_{0}}{R_{0}}$ = 0.0065ɛ^2^ + 1.57ɛ. Therefore, the corresponding derivative data (GF) followed a linear trend (GF = 0.013ε + 1.57) as shown in Figure S5. The sensor also showed a stable response to 60 stretching cycles, which demonstrated the eminent repeatability (Figure 8D).

### 2.5. Human Motion Detection

**Figure 9:**
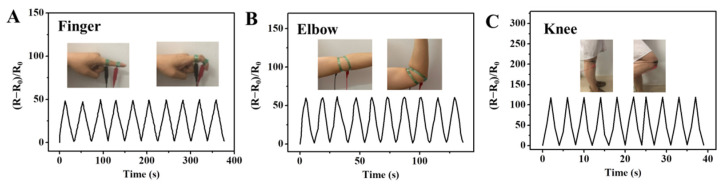
Monitoring of human body movements: (**A**) finger bending, (**B**) elbow bending, and (**C**) knee bending.

Additionally, the hydrogel sensor was validated to detect human motions, such as the flexing of a finger, an arm, and a leg (Figure 9). The bending movement caused the relative resistance to increase, while the straightening movement restored the sensor to the initial resistance. Compared with the motions of fingers and arms, the deformation degree of knee-bending was larger, resulting in a larger resistance change than others. Meanwhile, the hydrogel sensor responded very well to repeated joint-bending movements, indicating that the sensor had excellent reliability in human-motion monitoring.

### 2.6. Low-Temperature Strain Responsiveness

**Figure 10:**
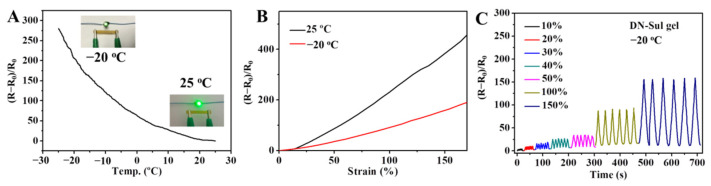
(**A**) The relative resistance change of the DN hydrogel with the temperature decreasing from 25 °C to −20 °C (the resistance at 25 °C was defined as R_0_). Inset pictures showed the bulb light’s variation in the circuit connected by the DN hydrogel at 25 °C and −20 °C. (**B**) The performance comparison of the DN hydrogel sensor at 25 °C and −20 °C. (**C**) Real-time relative resistance-change curves on multiple tensile strains at −20 °C.

As demonstrated, the DN hydrogel showed outstanding mechanical properties at sub-zero temperatures. Furthermore, the cryogenic sensitivity of the hydrogel was also investigated. An LED bulb connected to a circuit containing a piece of hydrogel was used to study the conductivity (Figure S6). As shown in Figure 10A, the conductivity of the hydrogel varied inversely with the temperature. When the temperature decreased, the migration of ions was impeded due to the limited movement of the polymer chains and the crystallization of water molecules. Figure 10B shows the response–strain curves of the gel sensors at 25 °C and −20 °C. Compared with the response value at normal temperatures, the response value at −20 °C was reduced to a certain extent. However, the gel sensor could still be utilized at low temperatures, as the response to 150% strain rose to 160%. Figure 10C illustrates the continuous response to the tensile strain ranging from 10% to 150% at −20 °C. The repeated curves at each strain demonstrated the excellent stability of the hydrogel sensor.

## 3. Conclusions

In conclusion, we prepared a DN hydrogel by immersing a CS/P(AA-co-AM) composite hydrogel into an Fe_2_(SO_4_)_3_ solution. Inorganic salt ions combined with the CS to form the energy-dissipative ionic network that enhanced the tensile property and conductivity simultaneously. Since the effective energy-dissipation mechanism was derived from the generated CS ionic network, the DN hydrogel exhibited prominent mechanical properties: tensile strength of 2.43 MPa, elastic modulus of 0.5 MPa, and toughness of 6.79 MJ/m^3^, as well as an outstanding self-recovery ability and excellent durability. Additionally, the introduction of ions provided the DN hydrogel with good freeze resistance and conductivity. Impressively, the strain sensor assembled from the DN hydrogel exhibited strain sensitivity and cycling stability. Even at low temperatures, the DN hydrogel still possessed good strain-sensing performance with slightly reduced sensitivity. The sensor also demonstrated a superior ability to detect various human body movements in real time. This study provides a new strategy for constructing functional hydrogel materials for stretchable, flexible sensors.

## 4. Materials and Methods

### 4.1. Materials

Acrylamide (AM, 98%), acrylic acid (AA, 99.5%), and methylene bisacrylamide (MBA, 98%) were bought from Alfa Aesar. Short-chain chitosan (CS, *M*w ≈ 10 kDa, degree of deacetylation > 90%) and iron sulfate hydrate were obtained from Aladdin Industrial Corp., China. The pure water was homemade by a water purification machine.

### 4.2. Fabrication of CS/P (AA-co-AM) DN Hydrogel

As shown in Figure 1, the DN hydrogel was fabricated by following two steps. First, the short-chain CS (0.75 g, 7.5 wt%), AM (2.13 g), AA (0.31 mL), MBA (0.16 mL), and Irgacure (54 mg) were added to pure water (10 mL) with continuous stirring. The homogeneous solution was then transferred into a glass mold and became CS/P (AA-co-AM) hydrogel under UV light (150 W) irradiation. Second, to get the DN hydrogel, the prepared CS/P (AA-co-AM) hydrogel was immersed in 2 M Fe_2_(SO_4_)_3_ solution for 30 min. A serial of DN hydrogels were prepared with different CS contents (0 wt%, 2.5 wt%, 5 wt%, 7.5 wt%, and 10 wt%).

### 4.3. Fabrication of the Hydrogel-Based Flexible Sensor

**Figure 11:**
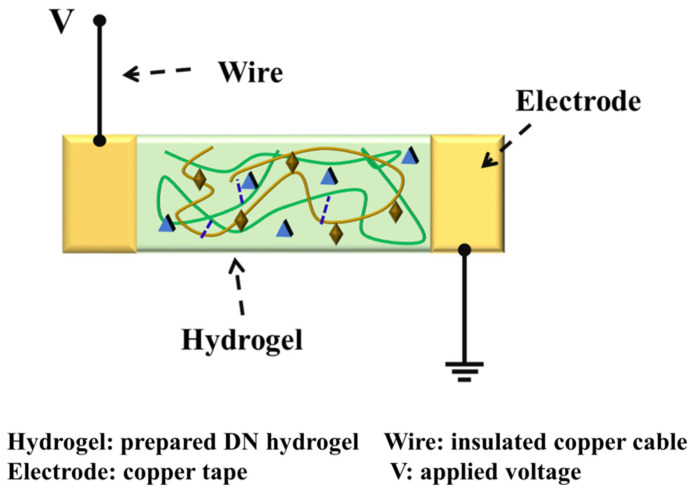
The schematic diagram of the strain sensor assembled using DN hydrogel and Cu electrodes.

The flexible sensor based on the DN hydrogel was assembled using two copper tapes attached at the DN hydrogel’s edges (Figure 11). The hydrogel-based flexible sensor was then sealed by a 3 M tape to lock the water in the hydrogel.

### 4.4. Characterization

The microtopography of the DN hydrogel was observed with a field-emission scanning electron microscope (FESEM, FEI Quanta 250 FEG). The hydrogel’s mechanical properties were measured by a universal tensile tester (MTS CMT4104) with hydrogel strips (25 mm × 3 mm × 1 mm) for an elongation test and hydrogel cylinders (diameter × height: 10 mm × 12 mm) for a compression test. According to the previous literature, the rates of the elongation test and compression test were 50 mm/min and 5 mm/min, respectively, while the load cells were 500 N and 10 kN, respectively [27,32]. The mechanical properties at low temperatures were measured with a universal tensile tester (MTS CMT4104) attached to a cryogenic box (MTS GDX200).

## Figures and Tables

**Table 1:** The detailed mechanical properties of the DN hydrogels with different CS contents.

CS Content	Elastic Modulus (MPa)	Fracture Strength (MPa)	Fracture Strain (%)	Toughness (MJ/m^3^)
0%	0.09	0.26	702	1.11
2.5%	0.17	0.73	623	2.24
5%	0.36	1.46	576	4.65
7.5%	0.50	2.44	539	6.80
10%	0.78	2.71	460	6.95

**Table 2:** The detailed mechanical properties of the DN hydrogels with different molar ratios of AA to AM.

n_(AA)_:n_(AM)_	Elastic Modulus (MPa)	Fracture Strength (MPa)	Fracture Strain (%)	Toughness (MJ/m^3^)
5%	0.29	1.24	629	4.35
10%	0.39	1.64	594	5.29
15%	0.50	2.44	539	6.79
20%	0.73	2.47	474	6.64
25%	0.80	2.63	433	6.26

## Data Availability

Not applicable.

## Supplementary Materials

The following supporting information can be downloaded at: https://www.mdpi.com/article/10.3390/gels8070424/s1, Figure S1: Mechanical properties of the composite hydrogel. (A) Compression. (B) Various tensile deformations: (B_1_) stretching, (B_2_) twisting–stretching, (B_3_ and B_4_) knotted–stretching, and (B_5_) crossing–stretching; Figure S2: (A) Elastic modulus. (B) Toughness of the composite and DN hydrogels calculated from the tensile curves; Figure S3: Pictures of the composite and DN hydrogels at −20 °C; Figure S4: (A) Stress–strain curves and detailed mechanical properties’ parameters of the composite hydrogel at different temperatures. (B) Tensile strength, (C) elastic modulus, and (D) elongation; Figure S5: GF variation of the DN hydrogel with strain; Figure S6: Circuit diagram of the lamp-lighting experiment.
