# Supplementary material for: A Highly Mechanical, Conductive, and Cryophylactic Double Network Hydrogel for Flexible and Low-Temperature Tolerant Strain Sensors
Source: Gels. 2022 Jul 7;8(7):424. doi: 10.3390/gels8070424 (PMC9322378; doi:10.3390/gels8070424)
Supplement: Supplementary file 1 [file gels-08-00424-s001.zip › gels-1776793-supplementary.pdf]

gels

Supplementary Materials

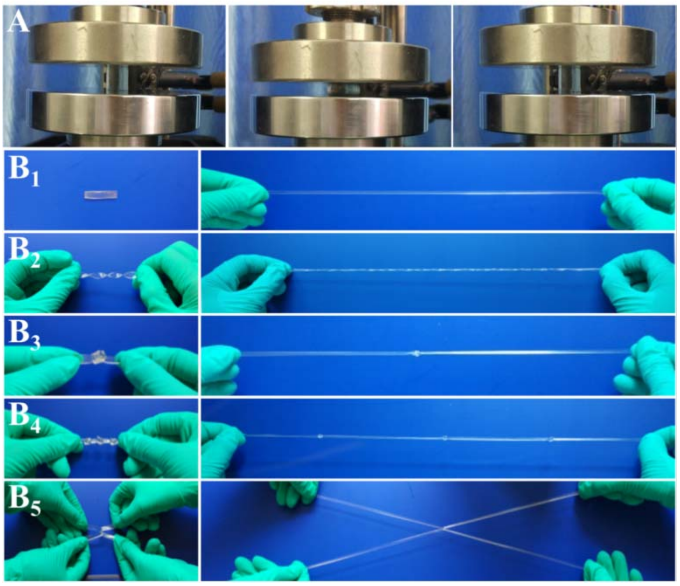

Figure S1. Mechanical properties of the composite hydrogel. (A) Compression, (B) Various tensile deformations: (B_1) stretching, (B_2) twisting-stretching, (B_3 and B_4) knotted-stretching and (B_5) crossing-stretching.

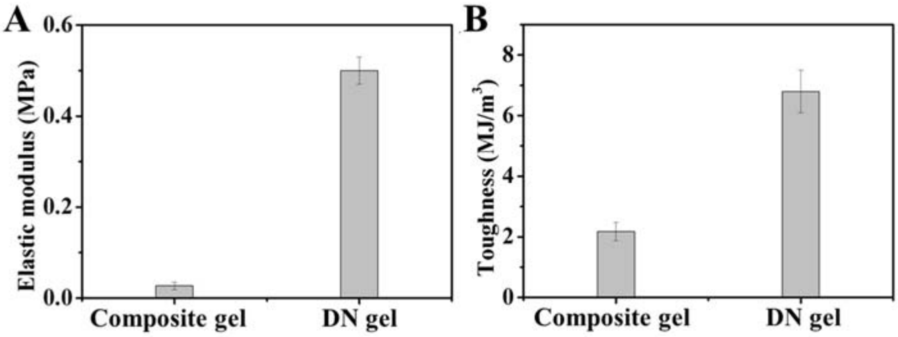

Figure S2. (A)Elastic modulus. (B) Toughness of the composite and DN hydrogels calculated from the tensile curves.

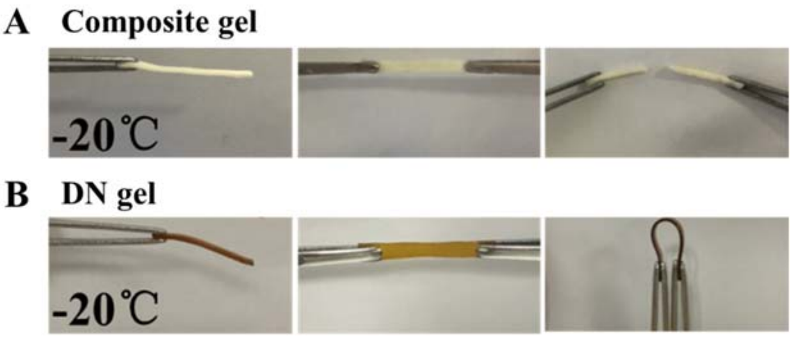

Figure S3. Pictures of the composite and DN hydrogels at -20 °C.

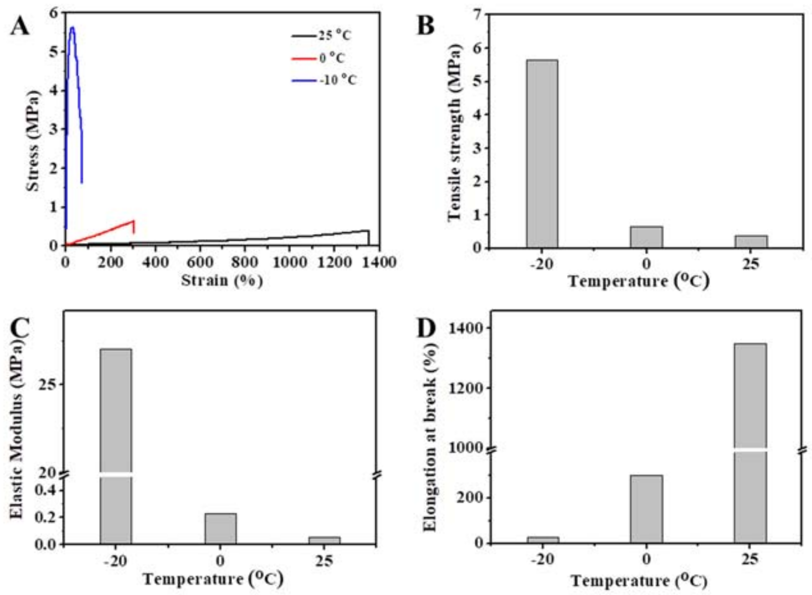

Figure S4. (A) Stress-strain curves and detailed mechanical properties' parameters of the composite hydrogel at different temperatures. (B) Tensile strength, (C) elastic modulus and (D) elongation.

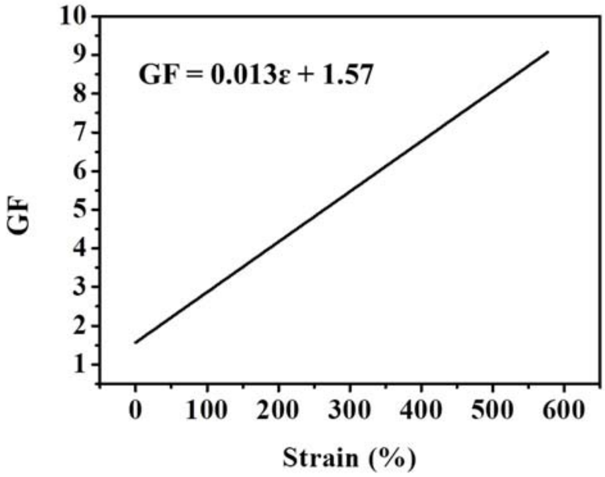

Figure S5. GF variation of the DN hydrogel with strain.

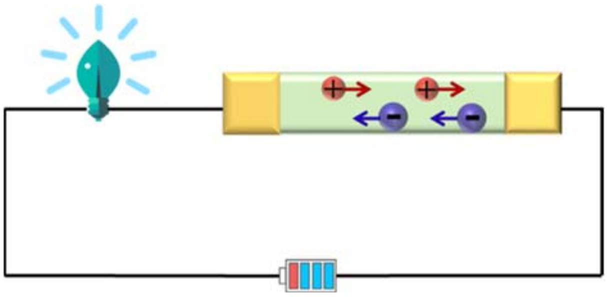

Figure S6. Circuit diagram of the lamp lighting experiment.
